# Distribution of *knock-down resistance *mutations in *Anopheles gambiae *molecular forms in west and west-central Africa

**DOI:** 10.1186/1475-2875-7-74

**Published:** 2008-04-29

**Authors:** Federica Santolamazza, Maria Calzetta, Josiane Etang, Elena Barrese, Ibrahima Dia, Adalgisa Caccone, Martin J Donnelly, Vincenzo Petrarca, Frederic Simard, Joao Pinto, Alessandra della Torre

**Affiliations:** 1 Istituto Pasteur-Fondazione Cenci-Bolognetti, Università "La Sapienza", Rome, Italy; 2 Dipartimento di Scienze di Sanità Pubblica, Sezione di Parassitologia, Università "La Sapienza", Rome, Italy; 3 Organisation de Coordination pour la lutte contre les Endémies en Afrique Centrale, Yaoundé, Cameroon; 4 Institut Pasteur, Dakar, Sénégal; 5 Department of Ecology and Evolutionary Biology, Yale University, New Haven, USA; 6 Vector Group, Liverpool School of Tropical Medicine, Liverpool, UK; 7 Dipartimento di Genetica e Biologia Molecolare, Università "La Sapienza", Rome, Italy; 8 Centro de Malaria e outras Doenças Tropicais, Instituto de Higiene e Medicina Tropical, Universidade Nova de Lisboa, Lisboa, Portugal

## Abstract

**Background:**

*Knock-down *resistance (*kdr*) to DDT and pyrethroids in the major Afrotropical vector species, *Anopheles gambiae *sensu stricto, is associated with two alternative point mutations at amino acid position 1014 of the voltage-gated sodium channel gene, resulting in either a leucine-phenylalanine (L1014F), or a leucine-serine (L1014S) substitution. In *An. gambiae *S-form populations, the former mutation appears to be widespread in west Africa and has been recently reported from Uganda, while the latter, originally recorded in Kenya, has been recently found in Gabon, Cameroon and Equatorial Guinea. In M-form populations surveyed to date, only the L1014F mutation has been found, although less widespread and at lower frequencies than in sympatric S-form populations.

**Methods:**

*Anopheles gambiae *M- and S-form specimens from 19 sites from 11 west and west-central African countries were identified to molecular form and genotyped at the *kdr *locus either by Hot Oligonucleotide Ligation Assay (HOLA) or allele-specific PCR (AS-PCR).

**Results:**

The *kdr *genotype was determined for about 1,000 *An. gambiae *specimens. The L1014F allele was found at frequencies ranging from 6% to 100% in all S-form samples (N = 628), with the exception of two samples from Angola, where it was absent, and coexisted with the L1014S allele in samples from Cameroon, Gabon and north-western Angola. The L1014F allele was present in M-form samples (N = 354) from Benin, Nigeria, and Cameroon, where both M- and S-forms were sympatric.

**Conclusion:**

The results represent the most comprehensive effort to analyse the overall distribution of the L1014F and L1014S mutations in *An. gambiae *molecular forms, and will serve as baseline data for resistance monitoring. The overall picture shows that the emergence and spread of *kdr *alleles in *An. gambiae *is a dynamic process and that there is marked intra- and inter-form heterogeneity in resistance allele frequencies. Further studies are needed to determine: i) the importance of selection pressure exerted by both agricultural and public health use of pyrethroid insecticides, ii) the phenotypic effects, particularly when the two mutations co-occur; and iii) the epidemiological importance of *kdr *for both pyrethroid- and DDT-based malaria control operations, particularly if/when the two insecticides are to be used in concert.

## Background

DDT and pyrethroids share the same mode of action on the insect nervous system, targeting the neuronal voltage-gated sodium ion channels. Molecular characterizations have revealed that various mutations in the S1-S6 transmembrane segments of domain II of the sodium ion channel gene give rise to resistance to these insecticides in a number of insect species [1-3]. In the major Afrotropical malaria vector, *Anopheles gambiae *sensu stricto (hereafter named simply *An. gambiae*), two point mutations at amino acid position 1014 of the voltage-gated sodium channel gene have been described, resulting in either a leucine-phenylalanine (L1014F) [2], or a leucine-serine (L1014S) [4] substitution. Both mutations have been shown to be linked with DDT and pyrethroid resistance phenotypes in field *An. gambiae *populations [2,4-9]. A relatively small number of studies have been conducted to determine the epidemiological impact of *kdr *target site insensitivity on the efficacy of pyrethroid and DDT based vector control [9-13]. Although a consensus has not been reached yet, some groups suggest that it may be associated with decreased efficacy of ITNs and pyrethroid based IRS [10,11]. In the latter case, programme staff were able to utilize carbamate insecticides, which have a different mode of action, and thereby overcome the resistance problem. Therefore, studies for determining the epidemiological impact and the extent of distribution of *kdr *mutations in *An. gambiae *populations are considered relevant for the design of insecticide-based control programmes and for informing the debate about the re-introduction of DDT into the vector control.

A number of studies, with limited geographical sampling, have detailed the distribution of *kdr *mutations in *An. gambiae*: most have either screened for the L1014F allele in west African countries [2,6,14-22], or the L1014S mutation in east Africa [4]. A few studies have screened for the presence of both resistance alleles in Nigeria [5], Cameroon [23], Equatorial Guinea [24], Gabon [25], Uganda [26], Kenya and Tanzania [27], Malawi and Mozambique [28].

The picture that emerges from these studies is that the *kdr *mutations are not homogeneously distributed in the two molecular forms of *An. gambiae *(termed M and S), which are considered as incipient species [29]. In fact in early studies, the absence of the L1014F allele in the M-form was considered one of the major pieces of evidence for a severe restriction of gene flow between the two forms [30]. To date, the L1014F mutation has been reported in several S-form populations from western and western-central Africa [6,18,30,31] and recently from Uganda [26]. The L1014S mutation, originally recorded in Kenya [4,27], has recently been found in populations from Uganda [26], Gabon [25], Cameroon [23] and Equatorial Guinea [24]. In most of the few M-form populations surveyed, *kdr *alleles were seldom found, even in areas where the mutations occur at high frequencies in sympatric S-form populations [5,16,20,22]. Where the L1014F allele was found in M-form individuals it was attributed to introgression between the two molecular forms [17,21,31,32], with the exception of two cases, Bioko Island (Equatorial Guinea) and Douala (Cameroon), where the L1014F mutation may have arisen independently in the M-form [19,23,33]. The L1014S allele has not been observed in the M-form although the number of individuals analysed was small [5,23,24].

This manuscript details the first effort to analyse the distribution of the L1014F and L1014S mutations in *An. gambiae *populations from Sub-Saharan Africa west of the Rift Valley. It is envisaged that the data will serve as baseline for both researchers and, more importantly, for control staff who manage the deployment of insecticides, the efficacy of which could be compromised by the presence of *kdr *alleles.

## Methods

### Sample collections

*Anopheles gambiae *samples were collected from 19 sites in 11 west and west-central African countries, between 1998 and 2006. For Kedougou (Senegal), two samples were analysed, collected in 2001 and 2005, respectively. Table 1 shows the sampling sites (listed from west to east and north to south), their geographical coordinates, dates and methods of collection, the ecology of the sampling sites and the total number of M and S molecular forms analysed for *kdr *genotype.

**Table 1:** Sampling sites (listed from west to east), geographical coordinates, dates and methods of collection, ecology of the sites and total number of *Anopheles gambiae *molecular forms genotyped for the *kdr *locus.

State	Site	Latitude	Longitude	Year of collection	Collection method	Ecology	S-form	M-form
SENEGAL	Kedougou	12°36'N	12°14'W	2001	IR-PSC	Sudan-Guinea savannah	39	0
	"	"	"	2005	IR-PSC	"	43	0
	Tambacounda	13°23N	13°44W	2006	IR-PSC	Sudan-Guinea savannah	2	22
THE GAMBIA	Maccarthy Island	13°31'N	14°46'W	2003	IR	Northern Guinea savannah	0	17
BURKINA FASO	Bobo Dioulasso	11°02'N	04°13'W	2001	LC	Sudan-savannah urban	24	31
GHANA	Accra area	05°38'N	00°15'E	2002	LC	Savannah/forest	25	0
IVORY COAST	Bouaké area	07°11' – 07°40'N	04°55' – 05°01'W	1998	IR	Southern Guinea savannah	23	0
BENIN	Dassa area	07°45'N	02°11'E	2002	IR	Southern Guinea savannah	48	5
NIGERIA	Kobape, Olugbo	07°00' – 07°20'N	03°00' – 03°30'E	2001	LC	Southern Guinea savannah, rural	49	41
CAMEROON	Mangoum	05°31' N	10°37'E	2006	LC	Tropical Mountain grassland, intensive agriculture	75	0
	Kribi	02°56'N	9°54'E	2005	LC	Humid Equatorial, coastal, urban	6	48
	Dabadi	4°35'N	13°41'E	2006	LC	Equatorial/tropical, urban	64	0
GABON	Benguia	01°37'S	13°26'E	1999	LC	Tropical humid, rural	57	0
ANGOLA	Cabinda	05°32'S	12°11'E	2003	IR-HC	Tropical humid	114	0
	Kikudo	06°07'S	12°22'E	2002	IR-PSC	Tropical humid	46	0
	Saurimo	09°39'S	20°23'E	2002	IR-PSC	Tropical humid	15	0
	Luanda area	08°50'S	13°14'E	2002–2003	IR-PSC, IR-HC, IR-NET	Tropical dry	0	42
	Namibe area	15°10'S	12°09'E	2002	IR-PSC, IR-HC, IR-NET	Desert	0	15
SÃO TOME and PRÍNCIPE	Riboque	00°19'N	06°43'E	2004	LC	Tropical humid, urban	0	100
	Rua Trabalhadores	01°38' N	07°25'E	1998	LC	Tropical humid, urban	0	33

IR-HC = hand-operated aspirators, indoor collection; IR-NET = indoor resting mosquitoes in bed-nets; IR-PSC = pyrethrum spray collection; LC = larval collection

### Molecular analyses

DNA was extracted by a variety of standard methods (e.g. phenol/chloroform, DNAzol kit by Molecular Research Center Inc. and Easy-DNA Invitrogen kit) and specimens were identified to molecular form by PCR-RFLP [34]. Samples were genotyped at the *kdr *locus either using the Hot Oligonucleotide Ligation Assay (HOLA) developed by Lynd *et al *[35] or by the Allele-Specific PCR (AS-PCR) [2,4].

A sub-sample of the HOLA-analysed sample had been previously genotyped by PCR for the L1014F allele [2] and the results of the two approaches were compared. When the results of the two approaches were not consistent, sequencing of a fragment of the domain II of the voltage-gated sodium channel gene containing the *kdr *locus was performed using standard primers Agd1 and Agd2 [2]. The PCR products were cleaned using a commercial kit (SureClean PCR Clean Up, BIOLINE) and bi-directionally sequenced.

### Statistical analyses

The maximum predicted frequency (y) for an allele to be present in a sample of a given size (x) but undetected in a sample was obtained from the upper 95% confidence limit of a binomial distribution, given by y = 1- 0.05^1/x^, following the example of Post and Millest [36].

## Results

Eight-hundred and two *An. gambiae *were *kdr *genotyped using the HOLA approach and 193 specimens from Gabon and São Tomé and Príncipe Islands were analysed by AS-PCR. The *kdr *genotype was successfully determined for more that 98% of the specimens.

Table 2 shows the *kdr *allele frequencies observed in the S-form samples (N = 628). The L1014F allele was present in all samples analysed, with the exception of samples from Kikudo (N = 46) and Saurimo (N = 15) in Angola. No difference in the L1014F frequency was observed between samples collected in Kedougou (Senegal) in 2001 and 2005 [chi-square = 0.57, degrees-of-freedom (df) = 1, P = 0.32]. The L1014S allele was found in samples from Cameroon, Gabon and north-western Angola (Cabinda). Observed genotypic frequencies were in agreement with the Hardy-Weinberg expectations in all samples with N>20.

**Table 2:** Frequencies of *kdr *alleles (and 95% confidence intervals, CIs) in S-form samples of *Anopheles gambiae *s.s.

State	Site	**N**	1014L [95% CI]	L1014F [95% CI]	L1014S [95% CI]
SENEGAL	Kedougou, 2001	39	85.9 [76.5–91.9]	14.1 [8.1–23.5]	0.0 [0.0–4.7]
	Kedougou, 2005	43	81.4 [71.9–88.2]	18.6 [11.8–28.1]	0.0 [0.0–4.2]
BURKINA FASO	Bobo Dioulasso	24	2.1 [0.4–10.9]	97.9 [89.1–99.6]	0.0 [0.0–7.4]
GHANA	Accra area	25	22.0 [12.8–35.2]	78.0 [64.8–87.3]	0.0 [0.0–7.1]
IVORY COAST	Toliak	23	0.0 [0.0–7.7]	100.0 [92.3–100.0]	0.0 [0.0–7.7]
BENIN	Dassa area	48	40.6 [31.4–50.6]	59.4 [49.4–68.7]	0.0 [0.0–3.9]
NIGERIA	Kobape, Olugbo	49	83.7 [75.1–89.7]	16.3 [10.3–24.9]	0.0 [0.0–3.8]
CAMEROON	Mangoum	75	0.7 [0.1–3.7]	84.0 [77.3–89.0]	15.3 [10.4–22.0]
	Kribi	6	25.0 [8.9–53.2]	66.7 [39.1–86.2]	8.3 [1.5–35.4]
	Dabadi	64	38.3 [30.3–46.9]	47.7 [39.2–56.3]	14.1 [9.1–21.1]
GABON	Benguia	57	86.8 [79.4.-91.9]	6.1 [3.0–12.1]	7.0 [3.6–13.2]
ANGOLA	Cabinda	114	51.3 [44.9–57.7]	18.0 [13.5–23.5]	30.7 [25.1–37.0]
	Kikudo	46	100.0 [96.0–100.0]	0.0 [0.0–4.0]	0.0 [0.0–4.0]
	Saurimo	15	100.0 [88.7–100.0]	0.0 [0.0–11.4]	0.0 [0.0–11.4]

1014L = insecticide-sensitivity allele: wild type; L1014F = insecticide-resistance allele: leucine-phenylalanine substitution at position 1014 of the voltage-gated channel gene; L1014S = insecticide-resistance allele: leucine-serine substitution. See text for details

All M-form samples genotyped (N = 354) were characterized by the exclusive presence of the wild-type 1014L allele, with the exception of the samples from Benin (Dassa, N = 5), Nigeria (Kobape, Olugbo, N = 41) and Cameroon (Kribi, N = 48), showing a frequency of 40.0% [95% Confidence Interval (CI) = 16.8–68.7], 19.5% (CI = 12.4–29.4) and 6.3% (CI = 2.9–13.0) of the L1014F allele, respectively.

In the two sampling sites where M and S forms were sympatric, L1014F frequencies in Nigeria were similar (16.3% in S and 19.5% in M-form) whilst higher frequencies were observed in the S-form in Cameroon (66.7% in S and 6.3% in M-form; Fisher's Exact Probability Test, P << 0.001).

The S-form specimens genotyped by both the HOLA and the PCR assay for the L1014F allele [30] were compared (N = 267). The results of the PCR genotyping were consistent with those of HOLA in 98% (125/127), 79% (27/34) and 89% (43/48) of 1014L/1014L, 1014L/L1014F and L1014F/L1014F genotypes, respectively. When the L1014S allele was present (sample from Cabinda, Angola), the PCR assay interpreted as 1014L/1014L all the 1014L/L1014S (31/31) and L1014S/L1014S (12/12) specimens, while L1014S/L1014F genotypes were interpreted either as L1014F/L1014F (13/15) or as 1014L/L1014F (2/15). Sequencing of the fragment of the domain II of the voltage-gated sodium channel gene containing the *kdr *codon from 18 of the specimens providing inconsistent results confirmed the interpretation obtained by HOLA, with a single exception (i.e. a 1014L/1014L HOLA-specimen which was 1014L/L1014F by both PCR and sequencing).

## Discussion

This study shows that *kdr *mutations are widespread in *An. gambiae *S-form in west and west-central regions of sub-Saharan Africa. The L1014F allele was constantly present from the western limits of the S-form geographic range in central Senegal to the sub-equatorial areas of north-western Angola. The L1014S allele was not found in S-form samples collected in western sampling sites (i.e. west of 10°W), while it was found together with the L1014F allele in western-central sites, in agreement with previous preliminary reports from Gabon [25], Cameroon [23], Equatorial Guinea [24], and Uganda [26]. Since no departures from Hardy-Weinberg expectations have been observed in these samples, the significant L1014F/L1014S heterozygote excess reported in Gabon by Pinto *et al *[25] could likely be a transient and/or localized phenomenon, rather than the result of a possible selective advantage of carriers of both mutations in the heterozygous state. Further surveys will be required to ascertain the epidemiological impact of the co-occurrence of the two *kdr *mutations, at the level of both the individual and population, in the west-central African region.

**Figure 1:**
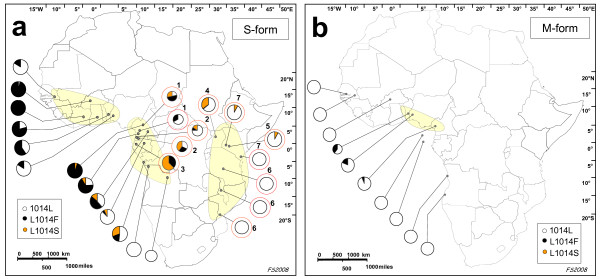
**Distribution of 1014L, L1014F and L1014S alleles in *Anopheles gambiae *S-form (1a) and M-form (1b) populations**. Smaller pie charts refer to collections with a sample size <20. Red circles refer to literature data reporting the results of the simultaneous genotyping of L1014F and L1014S alleles, per country, indicated by numbers as follows: 1 = Cameroon [23], 2 = Equatorial Guinea [24], 3 = Gabon [25], 4 = Uganda [26], 5 = Kenya [27], 6 = Kenya [43], 7 = Tanzania, Malawi, and Mozambique [28]. Shaded areas indicate zones with different patterns of *kdr *allele distribution. See text for details.

Merging the above results with all the data available to date on the simultaneous occurrence of the two *kdr *mutations in S-form [23-28], three main geographic areas can be distinguished based on *kdr *allele distribution and frequencies (Figure 1a): i) a western area, from Senegal to Nigeria, in which only the L1014F allele is present with frequencies >50% in most sites; ii) a west-central area from Cameroon in the north to Cabinda (Angola) in the south, extending eastward to Uganda, in which both *kdr *mutations are present at variable frequencies in most sites; iii) an area to the east of the Rift-Valley, where previous reports show L1014S frequencies below 10% in Kenya and where to the south both resistance mutations are likely absent.

The data obtained do not allow to determine whether the geographical differences in *kdr *allele frequencies among S-form populations is a result of non-uniform selective pressures due to variation in insecticide use, as recently hypothesized by Pinto *et al *[28]. However, it is interesting to note that the data from Angola suggest an impact of human activities on *kdr *distribution. All samples from Angola were characterized by the wild-type 1014L/1014L genotype, with the only exception of an S-form sample collected in Cabinda, less than 100 km apart from the sampling site of Kikudo [37]. Although it is not possible to completely rule out the presence of *kdr *alleles in the relatively small Kikudo sample (N = 46), as there is a 95% probability that an allele with a frequency of 3% or below would not be detected in the analysed sample (equivalent to mis-scoring one heterozygote genotype), the difference in the *kdr *allele frequencies between these sites is significant (chi-square = 67.9, df = 2, P < 0.001). This likely reflects the fact that the Kikudo area was involved in the civil war that ravaged Angola for the last 30 years, whilst Cabinda is located in an Angolan enclave within the Democratic Republic of Congo, which has been only marginally involved in the war, thus allowing for a larger scale use of pyrethroids against agricultural pests and/or malaria vectors [38].

This is the first regional survey of M-form specimens with respect to both resistance mutations. The results show the absence of the L1014S allele in all samples analysed and the presence of the L1014F allele in samples from a relatively restricted geographic region in the central part of Gulf of Guinea (i.e. Benin, Nigeria and Cameroon) (Figure 1b). Interestingly, the first report of the presence of L1014F in M-form was from Benin in 1998 [39], where it was shown to have introgressed from sympatric S-form populations [21] and where it has recently reached frequencies around 80% [15]. *Inter alia*, it can be hypothesized that the mutation is spreading from this region eastwards (i.e. Nigeria and later in Cameroon, where the L1014F frequency is 19% and 6%, respectively) and westwards in Burkina Faso [7]. Moreover, the hypothesis of a further "possible subdivision within the M molecular form" [40] may further complicate the picture.

Moreover, the different distribution of the two *kdr *mutations between sympatric M and S populations could also reflect different ecological/behavioural traits between M and S-forms, that might promote different exposure to insecticide selective pressures. For example, the M-form may be more adapted to urbanized, man-influenced ecological settings, whereas the S-form tends to prevail in rural settings [29,41], where a use of insecticides for agricultural purposes is expected to be greater.

From the technical point of view, the results highlight the high sensitivity and specificity of both the HOLA and AS-PCR approaches, which efficiently and simultaneously detected both *kdr *alleles, as already suggested on a smaller scale by Lynd *et al *[35] and Pinto *et al *[25]. Moreover, the results reinforce the notion that genotyping *kdr *alleles only with a single primer set (i.e. either the Martinez-Torres *et al *[2], or the Ranson *et al *[4] methods), is an incomplete and possibly misleading analysis. For a full description of *kdr *resistance in a given region, it is essential to screen for both *kdr *alleles.

## Conclusion

Although the results obtained refer to relatively few *An. gambiae *samples from a very large geographical range, they represent a first effort to analyse the overall distribution of the L1014F and L1014S mutations in sub-Saharan Africa, particularly west of the Rift Valley, where both molecular forms of this species co-occur in most of their range of distribution.

The results on S-form populations, combined with previously published data (Figure 1a), show a non-uniform distribution of *kdr *mutations throughout the continent: the L1014F is the only allele present west of 10°W latitude, while the two mutations co-exist in a wide geographic area in western-central Africa (ranging approximately from 6°N to 6°S).

The L1014F allele is present also in the M-form in an area comprised approximately between 5°W and 15°E, showing higher frequencies (>75%) in Benin and in the island of Bioko [15,33], and lower frequencies (<20%) in Burkina Faso, Nigeria and Cameroon. The absence of the L1014S allele in the M-form samples analysed will serve as baseline data for further studies on the possible occurrence and dispersal of this mutation in this form (Figure 1b).

The overall picture strongly suggests that the origin and spread of *kdr *alleles in *An. gambiae *is an ongoing process. There is evidence that both *kdr *alleles have originated in the S-form through multiple mutation events [28], whereas the L1014F allele may have arisen in the M-form either through introgression with the S-form or through independent mutation events [19,21,42]. Insect control activities, such as the large scale use of pyretroid insecticides for agricultural purposes, and possibly for domestic protection, may contribute largely to this process, as suggested by Pinto *et al *[28], who hypothesized a possible link between the recent expansion of cotton production in West Africa and an increased selective pressure due to pyrethroid insecticide use. This highlights the need to carefully monitor the evolution and spread of *kdr *target site insensitivity with a sensitive and precise method for the detection of both alleles and through more specifically designed spatial and temporal transects. Such surveys should be accompanied with the molecular analysis of non-target site resistance mechanisms for *An. gambiae*. This would facilitate an analysis of the relative contribution of *kdr *mutations into the resistance phenotype. Given the large geographic area where the two mutations co-occur in the S-form, it is also extremely important to undertake insecticide resistance surveys to correlate *kdr *genotypes to DDT and pyrethroid resistance phenotypes. This would allow to ascertain the phenotypic effect of both *kdr *mutations, particularly when co-occurring in the same individual, and to evaluate their possible effect on the efficacy of ITNs and pyrethroid based IRS, thus allowing control programme managers to make informed choices about the chemicals they use for vector control.

## Authors' contributions

FS carried out the molecular processing, participated in the analysis and interpretation of data, and contributed to the drafting of the manuscript; MC and EB helped with the molecular processing; JE and ID contributed to sample collections and molecular processing; AC, MJD and VP participated in the analysis and interpretation of data, and contributed to the drafting of the manuscript; FS and JP collected part of the samples and participated to the molecular processing and to the analysis and interpretation of data, and contributed to the drafting of the manuscript; AdT conceived and coordinated the study. All authors read and approved the final manuscript.
